# Development and Formulation of Nanofiber-Based Ophthalmic Inserts for the Treatment of Fungal Keratitis

**DOI:** 10.3390/pharmaceutics18040464

**Published:** 2026-04-10

**Authors:** Safaa Omer, Nándor Nagy, Júlia Pongrácz, Bence Dávid Tóth, Balázs Pinke, László Mészáros, Katalin Kristóf, Adrienn Kazsoki, Romána Zelkó

**Affiliations:** 1 University Pharmacy Department of Pharmacy Administration, Semmelweis University, Hőgyes Endre Street 7-9, H-1092 Budapest, Hungary; safaa.omer@phd.semmelweis.hu; 2 Department of Anatomy, Histology and Embryology, Semmelweis University, Tűzoltó Street 58, H-1094 Budapest, Hungary; nagy.nandor@semmelweis.hu; 3 Department of Laboratory Medicine, Faculty of Medicine, Semmelweis University, Üllői út 26, H-1085 Budapest, Hungary; pongracz.julia@semmelweis.hu (J.P.); kristof.katalin@semmelweis.hu (K.K.); 4 Department of Pharmaceutics, Semmelweis University, Hőgyes Endre Street 7, H-1092 Budapest, Hungary; toth.bence@semmelweis.hu; 5 Department of Polymer Engineering, Faculty of Mechanical Engineering, Budapest University of Technology and Economics, Műegyetem rkp. 3, H-1111 Budapest, Hungary; pinke.balazs.gabor@gpk.bme.hu (B.P.); meszaros.laszlo@gpk.bme.hu (L.M.)

**Keywords:** accelerated stability test, amphotericin B-loaded nanofibers, cytocompatibility study, fungal keratitis, in vitro antifungal efficacy, in vitro drug release study, ophthalmic insert

## Abstract

**Background/Objectives**: Fungal keratitis remains a vision-threatening infection, and current amphotericin B (AmphB) eye drops suffer from low corneal residence time, poor aqueous solubility, and the need for frequent dosing. This study develops electrospun nanofiber-based ophthalmic inserts combining polyvinyl alcohol (PVA), gamma-cyclodextrin (γ-CD), and sodium taurocholate (STC) to enhance AmphB solubility and provide a non-invasive, rapidly dissolving ophthalmic dosage form. **Methods**: γ-CD and STC-enhanced AmphB-loaded PVA nanofiber-based ophthalmic inserts with varying γ-CD and STC concentrations were prepared by electrospinning and characterized by scanning electron microscopy (SEM), Fourier transform infrared spectroscopy (FTIR), and X-ray diffraction (XRD). Drug content, in vitro release (Weibull modeling), antifungal activity against *Candida albicans*, *Fusarium solani*, and *Aspergillus fumigatus*, ocular cytocompatibility using the Hen’s Egg Test on Chorioallantoic Membrane (HET-CAM), and accelerated stability (40 ± 2 °C, 75 ± 5% relative humidity, 4 weeks) were evaluated. **Results**: Bead-free nanofibers with mean diameters between 216 ± 33 nm and 310 ± 35 nm were obtained, and XRD confirmed complete amorphization of AmphB within the PVA nanofiber matrix, forming an amorphous solid dispersion. All formulations showed rapid and nearly complete AmphB release (≈100% within 60 min), with Weibull β values < 0.75, indicating Fickian diffusion-controlled release. AmphB-loaded PVA nanofiber-based ophthalmic inserts produced inhibition zones and broth susceptibility profiles comparable to AmphB in dimethyl sulfoxide (DMSO), demonstrating preserved antifungal activity. HET-CAM scores (0–0.9) classified the inserts as practically non-irritant, and SEM/FTIR after accelerated storage showed no relevant morphological or physicochemical changes. **Conclusions**: These γ-CD and STC-enhanced AmphB-loaded PVA nanofiber-based ophthalmic inserts provide a non-invasive, rapidly dissolving ophthalmic dosage form that combines amorphous AmphB, immediate drug availability, and good ocular tolerance, supporting their further development as a patient-friendly treatment option for fungal keratitis.

## 1. Introduction

Fungal keratitis is a severe, life-threatening ocular infection primarily affecting the cornea and commonly caused by filamentous fungal species such as *Candida*, *Fusarium*, and *Aspergillus* [1]. The condition is associated with symptoms including ocular pain, conjunctival hyperemia, excessive lacrimation, purulent discharge, and visual impairment [2]. Predisposing factors for the development of fungal keratitis include corneal injury, contact lens wear, ocular surface disorders, the use of topical corticosteroids, and conditions that compromise the immune system [3]. If not adequately treated, fungal keratitis can progress to corneal perforation, stromal scarring, endophthalmitis, and ultimately blindness [4]. Current therapeutic strategies rely mainly on topical antifungal formulations; however, these approaches are limited by poor ocular bioavailability, rapid precorneal elimination, and the need for frequent administration, which may reduce patient compliance and therapeutic efficacy [5,6]. While invasive approaches such as intrastromal or intracameral injections may be employed, the development of innovative non-invasive drug delivery systems remains critically important [7].

Amphotericin B (AmphB) remains one of the most effective broad-spectrum antifungal agents for the treatment of fungal keratitis. Nevertheless, its clinical use in ophthalmic formulations is constrained by its extremely low aqueous solubility, instability in solution, and potential ocular toxicity at higher concentrations. These limitations necessitate the development of advanced drug delivery systems capable of improving AmphB solubility, stability, and ocular availability while minimizing adverse effects [8,9].

Cyclodextrins (CDs), particularly gamma-cyclodextrin (γ-CD), are widely used to enhance the solubility and stability of hydrophobic drugs through inclusion complex formation [10]. The large molecular dimensions of AmphB influence its binding affinity toward different CD types [11]. Due to its relatively large cavity size, γ-CD is especially suitable for accommodating bulky molecules such as AmphB, thereby increasing its solubility in aqueous environments [11]. Therefore, gamma-cyclodextrin (γ-CD), in particular, is used to improve the solubility of AmphB effectively more than other CDs [12,13]. The complexation process significantly increases the water solubility of AmphB, facilitating its incorporation into aqueous formulations without compromising its therapeutic efficacy [14]. Previous studies have demonstrated that γ-CD–AmphB inclusion complexes can improve the drug’s dissolution behavior and enable its formulation in aqueous media [13,14].

However, while cyclodextrins improve solubility, they do not necessarily enhance drug permeation across biological membranes. To address this limitation, bile salts such as sodium taurocholate (STC) can be incorporated as multifunctional excipients. STC possess surfactant properties that can further enhance the dissolution and absorption of poorly soluble drugs [15]. In ocular drug delivery, bile salts have been investigated as permeation enhancers capable of transiently modulating corneal epithelial tight junctions, thereby increasing paracellular transport of co-administered drugs [16]. Therefore, the combination of γ-CD and STC suggests synergistic effects on drug solubilization and membrane permeation [15,16]. However, the application of this combination strategy in electrospun ocular inserts for AmphB delivery remains unexplored.

Electrospun nanofiber-based (submicron-scale) systems have emerged as promising platforms for ocular drug delivery due to their high surface area-to-volume ratio, tunable morphology, and ability to incorporate both hydrophilic and hydrophobic drugs [17,18,19]. These systems can accommodate a range of therapeutic agents, from small molecules to macromolecules, and allow for the incorporation of multiple drugs within a single platform [20,21,22,23]. These systems can be engineered to provide either sustained or immediate drug release, depending on the polymer composition and structural design [24,25]. Nanofibrous inserts represent an attractive alternative to conventional eye drops, as they can increase precorneal residence time, reduce dosing frequency, and improve patient compliance without requiring invasive administration compliance [17,26,27,28]. Previous studies have demonstrated the potential of electrospun nanofibers in ocular therapy; however, many reported systems focus primarily on sustained release or polymeric optimization, with limited emphasis on simultaneous solubility enhancement and permeation improvement for poorly soluble drugs such as AmphB [29].

Selecting the appropriate material is crucial in the fabrication of nanofibers, as it directly influences their mechanical properties, biocompatibility, and drug-release behavior [30,31,32,33]. Polyvinyl alcohol (PVA) is widely employed in electrospinning due to its favorable properties, including biocompatibility, mechanical strength, processability, and the ability to modulate drug release. PVA-based electrospun fibers have been extensively characterized for ophthalmic applications, with studies demonstrating their suitability as rapidly dissolving or sustained-release platforms depending on crosslinking and formulation parameters [34,35,36,37].

Several studies have investigated electrospun fibers for ocular drug delivery. A comprehensive review by Omer and Zelkó (2021) summarized the scope of electrospun nanofiber-based ophthalmic inserts, highlighting their potential to overcome limitations of conventional eye drops [17]. More recently, Uzel et al. (2023) provided an overview of nanofibers in ocular drug targeting and tissue engineering, emphasizing the versatility of electrospun platforms for anterior segment [26].

Regarding AmphB, Lakhani et al. (2019) developed a γ-CD-based AmphB topical formulation with synergistic activity against fungal species, demonstrating the feasibility of γ-CD for AmphB solubilization [38]. Albadr et al. (2022) reported a rapidly dissolving microneedle patch for intracorneal AmphB delivery, representing an alternative minimally invasive approach [39]. However, to date, no study has combined γ-CD with a bile salt in an electrospun PVA-based insert designed for rapid dissolution and non-invasive ocular application for fungal keratitis.

Mucoadhesive ocular inserts have been developed using various cellulosic and synthetic polymers, as reviewed by Gupta et al. (2021), with emphasis on enhancing precorneal residence [28]. Similarly, Jawadi et al. (2022) discussed bio-inspired mucoadhesive polymers for drug delivery applications, providing context for the design of adherent ocular platforms [27]. To date, no study has reported the integration of γ-cyclodextrin (γ-CD) and a bile salt (sodium taurocholate, STC) within a rapidly dissolving electrospun polyvinyl alcohol (PVA)-based insert for non-invasive ocular delivery of amphotericin B (AmphB) in the treatment of fungal keratitis.

Amphotericin B-loaded in situ gelling electrospun nanofibers have been developed using strategies such as bile salt incorporation, PLGA nanoparticle encapsulation, and polyelectrolyte complex formation, with the latter demonstrating superior antifungal efficacy, stability, and reduced corneal toxicity compared to conventional eye drops [40].

Based on these considerations, we hypothesized that combining γ-CD and STC within electrospun PVA nanofibers may improve its solubility and stability, promote amorphization, and enable rapid drug release upon hydration while maintaining antifungal activity and acceptable ocular tolerability. In contrast to sustained-release systems, this study focuses on the development of a rapidly dissolving ophthalmic insert designed to provide immediate drug availability at the site of infection, which is particularly advantageous in acute conditions such as fungal keratitis. Collectively, these properties would provide a non-invasive, rapidly dissolving ophthalmic insert suitable for the treatment of fungal keratitis.

Therefore, the aim of this study was to develop and characterize γ-CD and STC-enhanced AmphB-loaded PVA nanofiber-based ophthalmic inserts with varying concentrations of γ-CD and STC; to evaluate their physicochemical properties by characterization of fiber morphology (SEM), chemical composition (FTIR), and solid-state properties (XRD); to quantify drug content and evaluate in vitro release behavior; to assess antifungal activity against *Candida albicans*, *Fusarium solani*, and *Aspergillus fumigatus* using agar diffusion and broth susceptibility assays; to determine ocular cytocompatibility using the Hen’s Egg Test on Chorioallantoic Membrane (HET-CAM); and to evaluate short-term accelerated stability under stressed conditions (40 ± 2 °C, 75 ± 5% relative humidity, 4 weeks). To the best of our knowledge, there is currently no nanofibrous ophthalmic insert specifically designed for AmphB in which γ-CD and a bile salt are jointly used to solubilize AmphB, promote amorphization, and support a non-invasive, rapidly dissolving ophthalmic dosage form for fungal keratitis as a step toward a clinically translatable, patient-friendly ocular drug delivery system.

## 2. Materials and Methods

### 2.1. Materials

Amphotericin B (AmphB) was obtained from Sigma-Aldrich Chemie GmbH (Schnelldorf, Germany). Polyvinyl alcohol (PVA, Mowiol^®^ 18–88 with an average molecular weight Mw ~130 kDa) was obtained from Merck Ltd. (Budapest, Hungary). Gamma-cyclodextrin (γ-CD, average molecular weight 1297.2 g mol^−1^) was purchased from Cyclolab Ltd. (Budapest, Hungary). Sodium taurocholate (STC, was the product of TCI Ltd. (Tokyo, Japan)). Dimethyl sulfoxide (DMSO ≥ 99.7%) and methanol (anhydrous, 99.8%) were provided by Merck Ltd. (Budapest, Hungary). Ethylenediaminetetraacetic acid (EDTA) was supplied by Molar Chemicals Ltd. (Budapest, Hungary). The distilled water of pharmacopeial grade was used for precursor solution preparation. All materials were used without any further purification processes.

### 2.2. Methods

#### 2.2.1. Phase Solubility Studies of Amphotericin B (AmphB)/Gamma-Cyclodextrin (γ-CD) Solution in Water

The solubility of AmphB in water was studied by adding excess AmphB to aqueous solutions containing various concentrations of γ-CD (25, 50, 75, 100, and 125 mg/mL). The mixtures were stirred for 3 h at 37 °C, followed by filtration through 0.45 µM filters (Millex^®^ hydrophobic PTFE syringe filter, Merck Ltd., Budapest, Hungary). The filtrates were appropriately diluted and analyzed using a UV–VIS spectrophotometer (Jasco 530, Jasco Inc., Easton, MD, USA) equipped with an inline probe. Absorbance was recorded at λ_max_ 407 nm, and AmphB concentration was determined using a previously validated calibration curve. All measurements were performed in triplicate, and mean values were reported [41].

For chemical stability assessment, a solution containing 0.15 mg/mL AmphB in the presence of 25 mg/mL γ-CD was prepared under the same conditions. The filtered solution was divided into four sealed vials and subjected to thermal treatment at 60 °C for 0, 30, 60, and 120 min under continuous stirring [42].

#### 2.2.2. Preparation of Electrospinning Solutions

By varying the concentrations of γ-CD and STC, nine different γ-CD and STC-enhanced AmphB-loaded PVA solutions were prepared (Table 1). These precursor solutions contain γ-CD and STC to enhance the solubility of AmphB, with PVA serving as the fiber-forming polymer. Each PVA solution was prepared by dispersing the specified amount of PVA in distilled water and subjected to stirring under heat until clear solutions were achieved. Subsequently, AmphB, γ-CD, and STC were added to the PVA solutions, which were then stirred at 37 °C for 3 h to ensure the complete solubilization of AmphB and the formation of a clear yellow solution.

#### 2.2.3. Electrospinning of the Solutions

The fabrication of γ-CD and STC-enhanced AmphB-loaded PVA nanofiber-based ophthalmic inserts was performed using a laboratory-scale electrospinning apparatus (SpinSplit Ltd., Budapest, Hungary). A 1 mL plastic Luer lock syringe (Sigma Aldrich Ltd., Budapest, Hungary) containing the precursor solution was connected to a conventional 22G needle via a Teflon tube (SpinSplit Ltd., Budapest, Hungary). The syringe was mounted on a pump to maintain a constant solution flow. Preliminary experiments were conducted to optimize the process parameters. During electrospinning, the flow rate was maintained at 0.08–0.1 μL/s, the applied voltage in a range of 10–20 kV, and the distance between the needle tip and the grounded collector was set at 10 cm, 12.5 cm, and 15 cm. Each sample was collected on aluminum foil wrapped around the grounded collector and subsequently stored in a desiccator for further analysis. Parameter combinations that yielded optimal sample morphology were used for further sample preparation. The process was carried out under ambient conditions of 22 ± 1 °C and 40 ± 5% relative humidity.

#### 2.2.4. Morphological Characterization

The electrospun samples were characterized morphologically utilizing a JEOL JSM-6380LA (Tokyo, Japan) scanning electron microscope (SEM). The samples were initially affixed to copper ingots with double-sided carbon adhesive and subsequently coated with gold under vacuum conditions. Imaging was conducted at magnifications of 3500× and 5000×, with a working distance of 10 mm and an accelerating voltage of 10 kV. The captured images were assessed for fibrous structures and the presence of beads. For the fibrous samples, the diameters of 100 randomly selected fibers (n = 100) were measured from two different images using ImageJ software (version: 1.54k, US National Institutes of Health, Bethesda, MD, USA). The average fiber diameters and their standard deviations were calculated using Excel 2010. Histograms were constructed using OriginPro 2018 software (v9.5.1., OriginLab Corporation, Northampton, MA, USA). To test the normality of the fiber diameter distribution, the skewness and kurtosis were calculated using Microsoft Excel 2010 functions.

The normality of fiber diameter distribution, skewness, and kurtosis were also calculated by using Microsoft Excel 2010 functions according to Equations (1) and (2):

(1) $$Skew=\frac{n}{\left(n-1\right)\left(n-2\right)}\Sigma ({\frac{xi-\bar{x}}{s})}^{2}$$

(2) $$Kurtosis=\frac{n}{\left(n-1\right)\left(n-2\right)\left(n-3\right)}\Sigma \left({\frac{xi-\bar{x}}{s})}^{4}\right\}-\frac{3{\left(n-1\right)}^{2}}{\left(n-2\right)\left(n-3\right)}$$

where *n* is the number of data points, $\bar{x}$ is the mean, and *s* is the standard deviation.

#### 2.2.5. Fourier Transform Infrared Spectroscopy (FTIR)

Fourier transform infrared spectroscopy (FTIR) was utilized to investigate the physicochemical characteristics, compatibility, and potential interactions between the polymers and various excipients, using a Jasco FT/IR-4200 spectrophotometer (Jasco Inc., Easton, MD, USA). The analysis included both the individual components and their corresponding fibrous blends. Spectral data were acquired over the range of 400–4000 cm^−1^ at a resolution of 4 cm^−1^, with measurements conducted at ambient temperature and averaged over 100 scans per sample.

#### 2.2.6. X-Ray Diffraction (XRD)

X-ray diffraction patterns were recorded using a PANalytical X’Pert3 Powder diffractometer (Malvern Panalytical B.V., Almelo, The Netherlands) equipped with Cu Kα radiation, operated at an accelerating voltage of 45 kV and an anode current of 40 mA. Data were collected over a 2θ range of 2–80°, employing a step size of 0.0080° and a counting time of 99.695 s per step in reflection mode, with continuous sample rotation at 1 s^−1^. The incident beam optics consisted of a programmable divergence slit configured to maintain a constant irradiated length of 15 mm, along with a fixed 2° anti-scatter slit. The diffracted beam path incorporated an X’Celerator Scientific ultra-fast line detector, a 0.02 soller slit, and a programmable anti-scatter slit set to ensure a constant observed length of 15 mm. Data acquisition was carried out using the PANalytical Data Collector Application (version 5.5.0.505; Malvern Panalytical B.V., Almelo, The Netherlands).

#### 2.2.7. Determination of the Drug Content

The AmphB content in each fibrous sample was quantified by weighing an amount equivalent to 15 µg (approximately 90 mg to 125 mg, depending on the specific formulation). These weighed samples were subsequently dissolved in 10 mL of phosphate buffer (pH 7.4) and stirred under ambient conditions for 30 min. The absorbance of the resulting solution was measured at a wavelength of λ_max_ 407 using a Jasco 530 UV–VIS spectrophotometer equipped with an inline probe. The concentration of AmpB was determined by measuring the absorbance at 407 nm, using a previously established calibration curve. The average concentration from three independent experiments was reported.

#### 2.2.8. In Vitro Release Study and Release Kinetics

The in vitro release of AmphB from electrospun nanofibers was evaluated using a modified dissolution approach, adapted from the basket method described in Pharmacopoeia Hungarica (Ph.Hg. VIII) [39]. This method was designed to simulate small-volume dissolution conditions, such as those encountered in the buccal cavity and ophthalmic sac. The dissolution study was performed in a 25 mL beaker. Samples containing 15 µg of AmphB were weighed, folded around a dry magnetic bar, and placed inside the beaker. The beaker was placed on a magnetic stirrer set to 100 rpm at a temperature of 35 ± 0.5 °C. To detect the absorbance of the released AmphB, an in-line probe of a Jasco-V-750 UV-VIS spectrophotometer (Jasco Inc., Easton, MD, USA) was immersed inside the beaker. A pre-heated dissolution medium (10 mL of phosphate buffer, pH 7.4, at 35 °C) was added to the beaker containing the sample, and absorbance measurements were taken at predetermined intervals (60 min) at λ_max_ 407 nm. All measurements were conducted in triplicate. The amounts of the AmphB released were quantified using a previously established calibration curve. The release profile was then constructed from the average values of these measurements. The kinetics of the drug release were then evaluated using the Weibull model according to Equation (3):

(3) $$M_{t}=M_{\infty }\left(1-e^{-\frac{{\left(t-t_{0}\right)}^{\beta }}{\tau_{d}}}\right)$$

where *M_t_* is the AmphB release at (*t*) time, while *M*_∞_ is the maximum amount of the released AmpB. The parameters *t*_0_ and *τ_d_* are the lag and average dissolution times, respectively. The shape parameter (β) of the Weibull function can indicate the mechanism of drug transport through the polymer matrix (*β* = 1, denotes first-order kinetics *β* ≤ 0.75, suggests Fickian diffusion, while 0.75 < *β* < 1, indicates a combination of Fickian diffusion and Case II transport, and *β* > 1 suggests complex release mechanisms) [40,41,42].

#### 2.2.9. Amphotericin B Nanofiber Activity Against *Candida albicans*, *Fusarium solani*, and *Aspergillus fumigatus* by Agar Diffusion Assay

Fungal suspensions of 0.5 McFarland density were prepared in saline from overnight cultures of *Candida albicans*, *Aspergillus fumigatus*, and *Fusarium solani*. The suspensions were plated on RPMI agar, and AmphB-loaded PVA nanofiber-based ophthalmic insert disks weighing 2.5 or 5 mg were placed on the surface of the plates. Sterile paper disks were used as negative controls, and for positive controls, 50 µL of 0.3 mg/mL AmphB solubilized in DMSO was added to the paper disks. Inhibition zones were measured after 24/48/72 h of incubation at 37 °C. The efficacy of the AmphB-loaded PVA nanofiber-based ophthalmic inserts was comparable to that of soluble AmphB.

#### 2.2.10. Amphotericin B Nanofiber Activity Against *Candida albicans*, *Fusarium solani*, and *Aspergillus fumigatus* by Broth Susceptibility Testing

Fungal suspensions of 0.5 McFarland density were prepared in saline from overnight cultures of *Candida albicans*, *Aspergillus fumigatus*, and *Fusarium solani*. 20 µL of the *C. albicans* suspension and 100 µL of *A. fumigatus* and *F. solani* suspensions were placed into 11 mL of RPMI broth. 1-1 mL of the inoculated RPMI broths were dispensed in sterile tubes. An AmphB-loaded PVA nanofiber-based ophthalmic insert disk (2.5 or 5 mg) was placed in each tube. 50 µL of 0.3 mg/mL AmphB in DMSO was added to tubes for control, and tubes containing only the fungi were used as growth controls. The tubes were incubated at 37 °C and checked for growth at 24/48/72 h.

#### 2.2.11. Cytocompatibility Study Using Hen’s Egg Test on Chorioallantoic Membrane (HET-CAM)

For the cytocompatibility study, the HET-CAM was employed as an alternative to animal testing for evaluating the irritation potential and biocompatibility of the developed γ-CD and STC-enhanced AmphB-loaded PVA nanofiber-based ophthalmic inserts. Freshly fertilized chicken eggs from White Leghorn chickens (*Gallus gallus domesticus*), obtained from a commercial supplier (Prophyl-BIOVO Hungary Ltd., Mohács, Hungary), were incubated at 37.5 ± 0.5 °C in a humidified HEKA 1+ egg incubator (Rietberg, Germany) for nine days to facilitate the development of the chorioallantoic membrane (CAM). This highly vascularized membrane is widely used as an experimental model for the evaluation of coagulation, inflammation, and irritation responses.

On the ninth day of incubation, the eggs were removed from the incubator, and the shell over the air sac was carefully perforated and enlarged to approximately 2 cm using ophthalmic surgical scissors. The inner shell membrane was then gently peeled away to expose the highly vascularized chorioallantoic membrane (CAM).

The test samples (AmphB-loaded nanofiber mats and corresponding neat fibers, each weighing 90 mg) were carefully placed onto the CAM surface, and the tissue response was monitored for 5 min. During this period, the CAM was examined for indications of irritation, including hyperemia, hemorrhage, and coagulation. These effects were assessed based on both their onset time and severity [43,44].

The obtained results were evaluated against a negative control (phosphate-buffered saline (PBS), pH 7.4) and a positive control (0.1 N NaOH solution) to confirm the validity and reliability of the assay. Images for the HET-CAM test were captured at 0.5, 2, and 5 min using a Nikon SMZ25 stereomicroscope (Unicam Ltd., Budapest, Hungary). The acquired images were subsequently processed with Nikon’s proprietary software, QCapture Pro 2 (NIS-Elements Basic Research version; AURO-SCIENCE Consulting Ltd., Budapest, Hungary).

#### 2.2.12. Accelerated Stability Study

An accelerated stability study (40 ± 2 °C, 75 ± 5% relative humidity, 4 weeks) was performed under elevated stress conditions to predict potential time-dependent changes in the developed γ-CD- and STC-enhanced AmphB-loaded PVA nanofiber-based ophthalmic inserts. For this purpose, samples were wrapped in aluminum foil and sealed in hermetic zip-lock bags. All samples were prepared under identical conditions and appropriately labeled prior to exposure to the stress environment. The samples were stored in a stability chamber (Sanyo type 022, Sanyo, Leicestershire, UK) maintained at 40 ± 2 °C and 75 ± 5% relative humidity for a duration of 4 weeks. Stability was monitored at predetermined time points (0, 1, 2, 3, and 4 weeks). At each interval, the samples were evaluated for morphological and physicochemical changes using scanning electron microscopy (SEM) and Fourier transform infrared spectroscopy (FTIR), respectively.

#### 2.2.13. Statistical Analysis

OriginPro 2018 software (v9.5.1., OriginLab Corporation, Northampton, MA, USA) was used for figures construction and statistical analysis of the data. Additionally, Microsoft Excel 2010 functions were utilized to evaluate the normality, as well as the skewness and kurtosis, of the fiber diameter distribution.

## 3. Results

### 3.1. Solubility Study of Amphotericin B (AmphB)/Gamma-Cyclodextrin (γ-CD)

The solubility of AmphB was measured in aqueous solutions in the presence of γ-CD at different concentrations. Increasing γ-CD concentration in the solution resulted in a significant increase in the solubility of AmphB. A phase solubility diagram was constructed by plotting the molar concentration of dissolved AmphB against the concentration of γ-CD (Figure S1). The resulting curve showed a linear increase in AmphB solubility with increasing γ-CD concentration up to a certain limit. The linear portion of the plot suggests the formation of a 1:1 molar ratio complex between AmphB and γ-CD, following an AL-type (linear) phase solubility profile [45].

The apparent stability constant (K1:1) for the AmphB/γ-CD complex was calculated using the slope of the linear portion of the phase solubility diagram (Y = 0.0097x − 0.0655) and Equation (4):

(4) $$k_{1:1}=\frac{Slope}{s_{0}\left(1-Slope\right)}$$

K1:1 is the apparent stability constant (M^−1^), S0 is the intrinsic solubility of AmphB in water and slope is the slope of the straight line of the phase solubility diagram calculated using linear regression. The calculated value of K1:1 was 3202 M^−1^, and the slope < 1 (0.0097).

Temperature stability studies revealed that γ-CD helps maintain AmphB stability at both room and elevated temperatures, with AmphB retaining >95% of initial concentration after 120 min at 60 °C (Table S1).

### 3.2. Morphological Features

**Figure 1:**
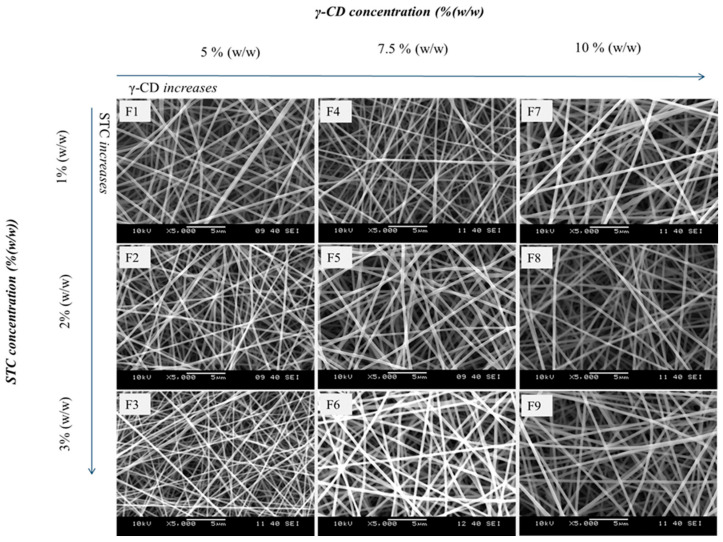
Scanning Electron Microscope (SEM) image of amphotericin B-loaded (AmphB-loaded) nanofibers. Samples prepared from AmphB/gamma-cyclodextrin (γ-CD)/polyvinyl alcohol (PVA)/sodium taurocholate (STC). (magnification: 5000×). All samples contained PVA (12% *w*/*w*) and AmphB (0.03% *w*/*w*). (**F1**–**F3**) 5% γ-CD with 1–3% STC; (**F4**–**F6**) 7.5% γ-CD with 1–3% STC; (**F7**–**F9**) 10% γ-CD with 1, 2 and 3% STC.

The SEM images showing the morphological characterization of electrospun AmphB-loaded samples are presented in Figure 1. The SEM images revealed bead-free, randomly oriented fiber orientation.

To assess the fiber diameter and uniformity of fiber distribution, average diameter, skewness, and kurtosis were calculated. The average fiber diameter range was approximately 216 ± 33 nm to 310 ± 35 nm, which is within the nanometer scale range, ensuring the fast and complete drug release. The values of skewness and kurtosis reveal that the distribution curves of electrospun AmphB-loaded samples are of normal and skewed shapes (Table S2).

In conclusion, the results indicate that different solutions’ compositions produced different distribution curves, including homogenous normal distribution and compound distribution curves (Figure S2). The appropriate solution composition does not guarantee a homogenous normal distribution curve because many factors overlap during electrospinning, including, for instance, the composition of the precursor solution as well as the process parameters. Therefore, electrospun nanofiber images should be further analyzed to evaluate normally distributed curves from compound distribution curves using different mathematical tools such as skewness and kurtosis [46,47].

### 3.3. Fourier Transform Infrared Spectroscopy Spectral Analysis

**Figure 2:**
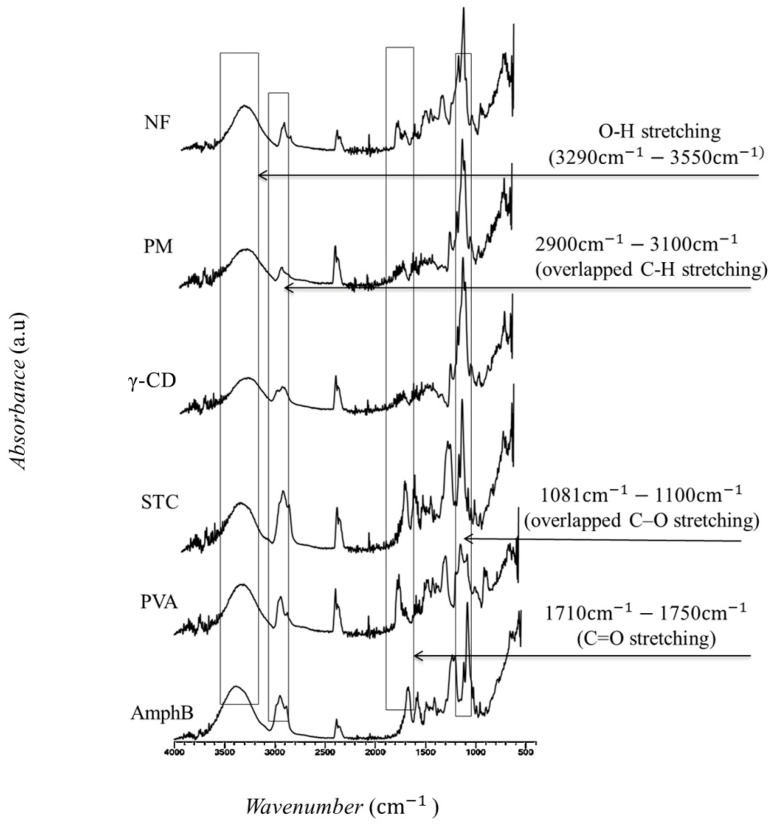
Fourier transform infrared (FTIR) spectra of the amphotericin B-loaded (AmphB-loaded) fibers. Where AmphB: amphotericin B; PVA: (polyvinyl alcohol, Mw ~130 kDa); STC: sodium taurocholate; γ-CD: gamma-cyclodextrin; PM: physical mixture; NF: nanofibers.

The FTIR analysis was performed to evaluate the chemical composition and interactions between AmphB, γ-CD, PVA, and STC within the nanofiber. The FTIR spectra for each component, the physical mixture, and the final nanofiber formulation were analyzed to identify the functional groups and changes in characteristic peaks (Figure 2).

The FTIR spectrum of pure AmphB showed characteristic peaks at 3430 cm^−1^, corresponding to O-H stretching vibrations, 2923 cm^−1^ for C-H stretching, and a strong peak at 1650 cm^−1^, indicative of the amide band (C=O stretching) [48]. The FTIR spectrum of γ-CD exhibited characteristic peaks at 3380 cm^−1^ corresponding to O-H stretching vibrations, 2925 cm^−1^ for C-H stretching, and a characteristic peak at 1030 cm^−1^ was attributed to C-O stretching [49,50].

The FTIR spectrum of PVA displayed characteristic peaks at 3300 cm^−1^ for O-H stretching, 2940 cm^−1^ corresponding to C-H stretching, and a peak at 1730 cm^−1^ associated with C=O stretching of the acetate group present in partially hydrolyzed PVA. The peak at 1090 cm^−1^ was due to C-O stretching vibrations [51]. The FTIR spectrum of STC showed characteristic peaks at 3420 cm^−1^ for O-H stretching, 2930 cm^−1^ for C-H stretching, and a sharp peak at 1640 cm^−1^ indicative of C=O stretching vibrations [52]. The FTIR spectrum of γ-CD exhibited characteristic peaks at 3380 cm^−1^ corresponding to O-H stretching vibrations, 2925 cm^−1^ for C-H stretching, and a characteristic peak at 1030 cm^−1^ was attributed to C-O stretching [53,54].

The FTIR spectrum of PVA displayed characteristic peaks at 3300 cm^−1^ for O-H stretching, 2940 cm^−1^ corresponding to C-H stretching, and a peak at 1730 cm^−1^ associated with C=O stretching of the acetate group present in partially hydrolyzed PVA. The peak at 1090 cm^−1^ was due to C-O stretching vibrations [55]. The FTIR spectrum of STC showed characteristic peaks at 3420 cm^−1^ for O-H stretching, 2930 cm^−1^ for C-H stretching, and a sharp peak at 1640 cm^−1^ indicative of C=O stretching vibrations [56]. The FTIR spectrum of the physical mixture and nanofibers of all components (AmphB, γ-CD, PVA, and STC) demonstrated the presence of all characteristic peaks corresponding to the individual components. This suggests that the formulation process did not induce any significant chemical changes in the physical mixture and the formulation.

### 3.4. X-Ray Diffraction (XRD) Analysis

**Figure 3:**
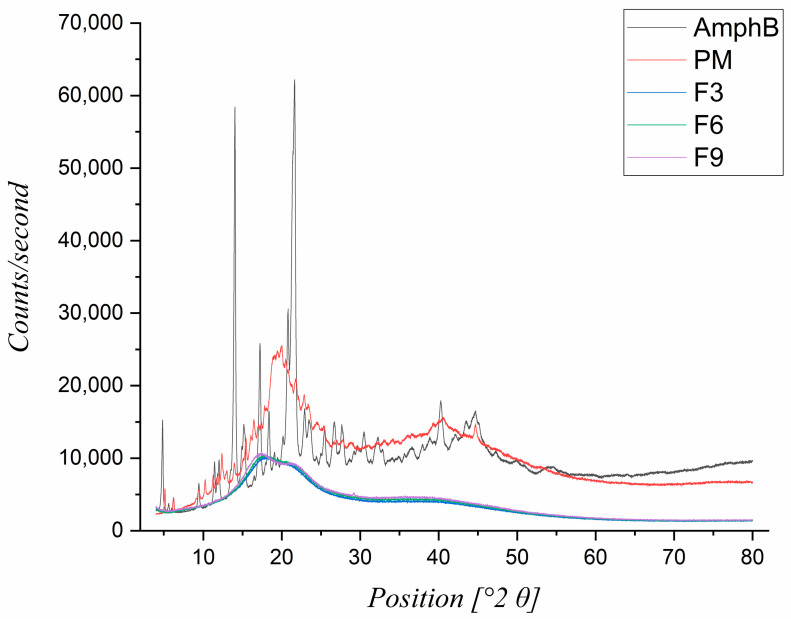
X-ray Diffraction (XRD) pattern of amphotericin B-loaded (Am-phB-loaded) nanofibers. The diffraction peaks correspond to the crystalline AmphB, physical mixture (PM), and AmphB-loaded nanofibers (F3, F6, and F9). The components for PM, F3, F6, and F9 are AmphB/gamma-cyclodextrin (γ-CD)/polyvinyl alcohol (PVA)/sodium taurocholate (STC). F3 has 5% γ-CD and 3% STC, F6 has 7.5% γ-CD and 3% STC, and F9 has 10% γ-CD and 3% STC. The reduced or absent crystalline peaks in the nanofibers indicate amorphization of AmphB. The XRD analysis confirms the successful amorphization of crystalline AmphB.

XRD analysis was carried out to investigate the crystalline structure of the AmphB compared to its physical mixture, and the nanofiber formulation that is composed of AmphB, γ-CD, PVA, and STC (Figure 3).

The XRD pattern of pure AmphB exhibited distinct, sharp peaks, indicative of its crystalline nature. In contrast to pure AmphB, the physical mixture showed a reduction in the AmphB peak intensities compared to the pure AmphB peak. The presence of this peak suggests that AmphB still retained some degree of crystallinity within the physical mixture. However, the disappearance of these peaks indicates the formation of amorphous solid dispersion (ASD) from crystalline AmphB. These results indicate that the electrospinning technique induces an amorphization process. This amorphous form of AmphB is beneficial in terms of higher solubility and faster dissolution rates compared to the crystalline AmphB.

### 3.5. Drug Content Analysis

The drug content in the nanofibers was determined to ensure uniform distribution and appropriate dosage of the AmphB within different electrospun AmphB-loaded formulations. The mean drug content was found to be 100 (% (*w*/*w*)). The results demonstrated consistent drug content across different nanofiber samples. These results indicate the successful incorporation of the drug during the electrospinning process. Furthermore, the presence of γ-CD in the formulation enhances the solubility of AmphB. This enhancement in the solubility maintained the AmphB in a more soluble state, thus facilitating its incorporation into the nanofibers.

### 3.6. In Vitro Release Study

**Figure 4:**
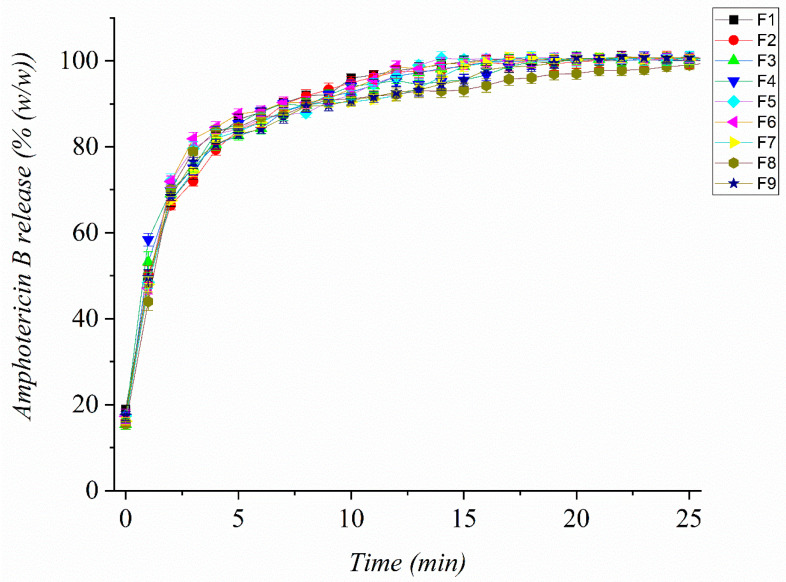
In vitro dissolution profile of Amphotericin B-loaded (AmphB-loaded) nanofibers. The release of AmphB from the nanofiber matrix was measured over time in a phosphate-buffered solution (pH 7.4) at 35 ± 0.5 °C. All formulations contained PVA (12% *w*/*w*) and AmphB (0.03% *w*/*w*); γ-CD was 5% (F1–F3), 7.5% (F4–F6), or 10% (F7–F9), and STC ranged from 1% to 3% within each group.

The in vitro release study of electrospun AmphB-loaded nanofibers demonstrated a rapid and complete drug release profile (Figure 4).

All formulations exhibited rapid and nearly complete AmphB release (≈100% within 60 min). This rapid release could be attributed to the high surface area-to-volume ratio of the nanofibers, which facilitates faster drug dissolution. Upon immersion in the release medium, the nanofiber matrix quickly hydrated, allowing the drug to dissolve rapidly. This is consistent with the properties of nanofibrous materials, where the small diameter of the fibers contributes to an enhanced release rate.

To understand the release mechanism of AmphB from the nanofibers, the in vitro release data were fitted to the Weibull distribution model since it can be applied to different release behaviors. All formulations from the various bases were successfully fitted to the Weibull model (R2 > 0.9), indicating linear regression, and the release parameters β values for all formulations were <0.75, indicating Fickian diffusion (Table 2). This suggests that the diffusion process primarily governs the drug release from the nanofibers. This immediate drug availability at the site of infection could potentially lead to improved clinical outcomes by quickly achieving therapeutic drug concentrations in the corneal tissue.

### 3.7. Amphotericin B Nanofiber Activity Against Candida albicans, Fusarium solani, and Aspergillus fumigatus

#### 3.7.1. Agar Diffusion Assay

The antifungal activity of the developed γ-CD and STC-enhanced AmphB-loaded PVA nanofiber-based ophthalmic inserts was assessed against *Candida albicans*, *Fusarium solani*, and *Aspergillus fumigatus* using an agar diffusion assay, and the results were compared with those obtained from the AmphB solution in DMSO. The agar diffusion assay revealed that there were no significant differences in the antifungal activity of AmphB when delivered via nanofibers or a standard solution of AmphB in DMSO (Figure S3). The zones of inhibition (ZOI) for the different fungal strains are summarized in (Table 3). The comparable activity of AmphB-loaded nanofibers to the AmphB solution in DMSO indicates that the encapsulation of AmphB within nanofibers does not compromise its antifungal effectiveness.

#### 3.7.2. Broth Susceptibility Testing

**Figure 5:**
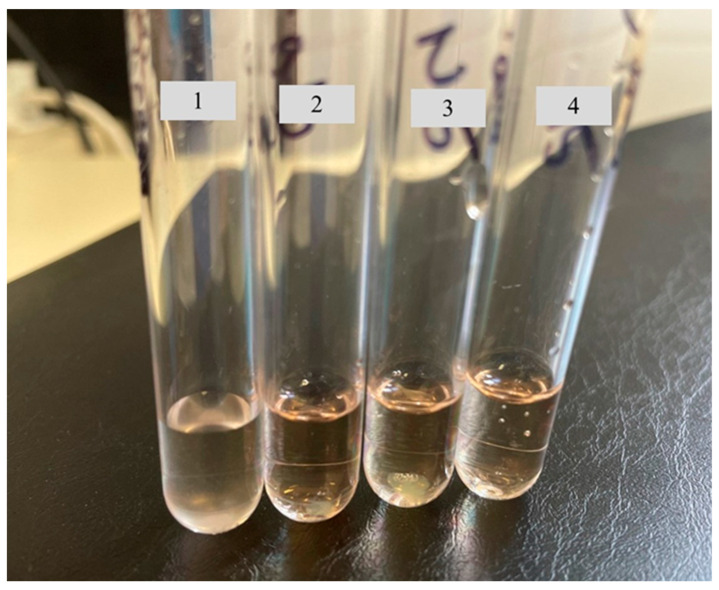
Broth susceptibility test for comparative antifungal activity of the amphotericin B-loaded (AmphB) nanofiber and the Amph B solution in Dimethyl sulfoxide (DMSO) against *Candida albicans*. Tube 1: growth control; Tube 2: AmphB-loaded Nanofiber 2.5 mg; Tube 3: AmphB-loaded Nanofiber 5 mg; Tube 4: AmphB solution in DMSO).

The results from the broth susceptibility testing are presented in Figure 5. The results demonstrated that the AmphB-loaded fibers and AmphB solution in DMSO were effective in killing the fungi after incubation compared to the control tube, which does not contain AmphB in any form. The results reveal that the AmphB-loaded nanofibers possess antifungal activity comparable to the AmphB solution in DMSO. This finding indicates that the novel nanofiber formulation retains the therapeutic efficacy of AmphB against all three fungal strains, and embedding the drug within the nanofibers did not compromise its antifungal efficacy. Therefore, this non-invasive, rapidly dissolving ophthalmic dosage form could be a promising alternative for the treatment of fungal keratitis.

### 3.8. Cytocompatibility Study

The cytocompatibility of the fibrous samples was evaluated using the HET-CAM assay, a reliable alternative to in vivo tests used to assess irritation potential and biocompatibility. The study was performed to ensure that the developed formulations are non-irritant after ophthalmic application.

**Figure 6:**
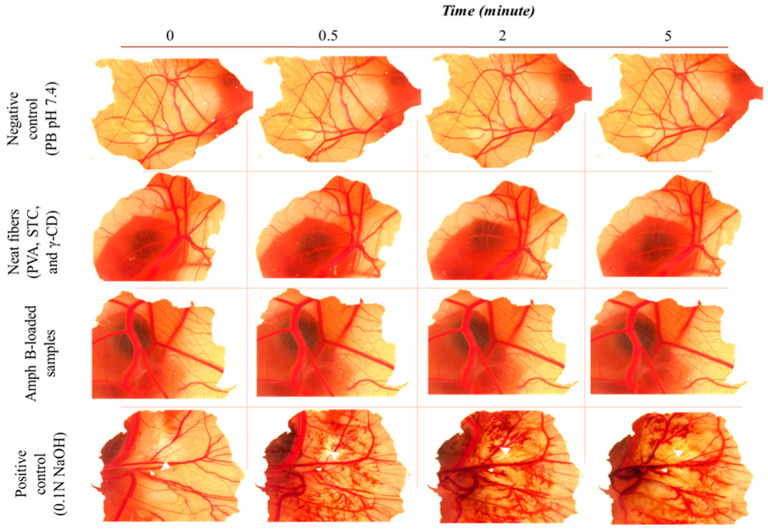
Representative images of Hen’s Egg Test on Chorioallantoic Membrane (HET-CAM). The test was conducted on the vascular structure of the CAM on day 9 of chicken development for a period of 5 min for electrospun amphotericin B-loaded (AmphB-loaded). The figure illustrates the comparative analysis of the irritation potential of the fibrous samples (neat and Am-phB-loaded) against the controls (phosphate-buffered saline (PBS) as a negative control and 0.1 N NaOH as a positive control). A strong hemorrhage induced by 0.1 N NaOH placed on the surface of a chick CAM at embryonic day 9.

The result of the HET-CAM assay is presented in Figure 6. The results demonstrate that the γ-CD and STC-enhanced AmphB-loaded PVA nanofiber-based ophthalmic inserts are practically non-irritant at the loaded dose, as indicated by the absence of significant redness, hemorrhage, or coagulation on the chorioallantoic membrane of fertilized 9th-day chicken embryos. Quantitatively, the irritation scores fall within the 0–0.9 range, which, according to the standard classification scale (Table 2) [44], categorizes the samples as practically non-irritant, suggesting the cytocompatibility of the samples and suitability for ocular use.

### 3.9. Accelerated Stability Investigation

**Figure 7:**
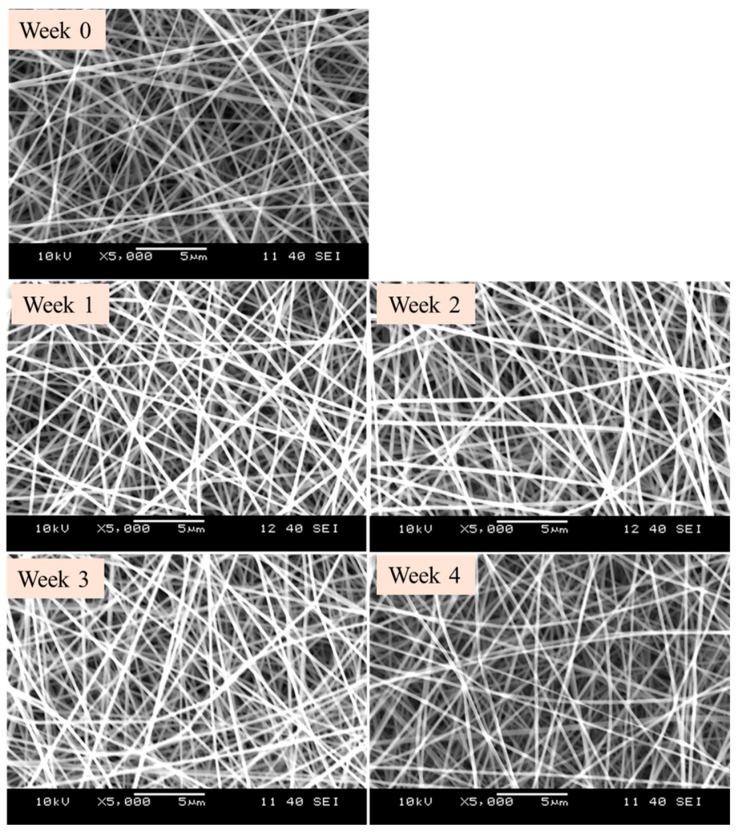
Scanning electron microscopic (SEM) images of amphotericin B-loaded (AmphB-loaded) fibers subjected to accelerated stability study. Samples were stored under stress conditions (40 ± 2 °C, 75 ± 5% relative humidity) for 0, 1, 2, 3, and 4 weeks. (magnification: 5000×).

The results of the accelerated stability study indicate that the fibrous samples exposed to elevated humidity and temperature are stable and maintain their structural and chemical integrity over the four weeks. The SEM im-ages confirm that the nanofibers retain their structural integrity throughout the stability study period (0, 1, 2, 3, and 4 weeks) (Figure 7). The nanofiber structure remains intact with no noticeable degradation, suggesting that there are no significant physical changes or degradation of the nanofibers under accelerated stability conditions (40 ± 2 °C and 75 ± 5% relative humidity).

**Figure 8:**
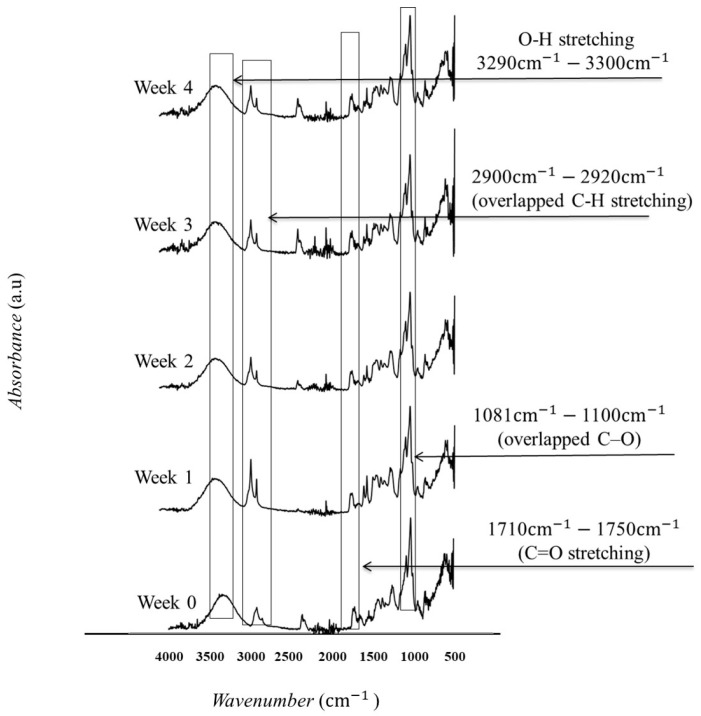
Fourier transform infrared (FTIR) spectra of the amphotericin B -B-loaded (AmphB-loaded) fibers subjected to accelerated stability study. Samples were stored under stress conditions (40 ± 2 °C, 75 ± 5% relative humidity) for 0, 1, 2, 3, and 4 weeks.

The FTIR spectra of the samples before and after the stability study are shown in (Figure 8). The FTIR spectrum obtained after the accelerated stability study shows that the characteristic peaks are retained, suggesting that there have been no significant chemical changes or interactions affecting the drug or polymer matrix. The spectra confirm that the solid-state properties of the formulation remain stable over the study period. This stability is crucial for ensuring that the inserts provide consistent and reliable delivery of the Am-phB over their intended use period.

## 4. Conclusions

In this study, electrospun PVA-based ophthalmic fiber inserts loaded with Amphotericin B (AmphB) were successfully developed using γ-cyclodextrin (γ-CD) and sodium taurocholate (STC) as functional excipients. The proposed system represents a promising, non-invasive, rapidly dissolving ophthalmic dosage form for the management of fungal keratitis, effectively addressing the major limitations of AmphB, including poor aqueous solubility and low ocular bioavailability.

The combination of polyvinyl alcohol (PVA), γ-CD, and STC enabled efficient incorporation of AmphB into a submicron fibrous matrix while enhancing its apparent solubility. The electrospun fibers exhibited uniform, bead-free morphology with diameters ranging from 216 ± 33 nm to 310 ± 35 nm. X-ray diffraction confirmed the transformation of AmphB into an amorphous state within the fibers, which is expected to contribute to improved dissolution behavior.

All formulations demonstrated rapid and nearly complete drug release (≈100% within 60 min). The release kinetics, characterized by Weibull β values < 0.75, indicated a Fickian diffusion-controlled mechanism, supporting immediate drug availability at the site of infection. This rapid-release profile is particularly advantageous for acute ocular conditions such as fungal keratitis, where prompt therapeutic action is required, distinguishing the system from conventional sustained-release approaches.

The antifungal activity of the developed inserts was preserved, showing comparable efficacy to AmphB in dimethyl sulfoxide against *Candida albicans*, *Fusarium solani*, and *Aspergillus fumigatus*, confirming that incorporation into the fibrous matrix does not compromise drug performance.

Biocompatibility assessment using the HET-CAM assay classified the formulations as practically non-irritant (irritation score: 0–0.9), indicating favorable short-term ocular tolerability. However, although the HET-CAM assay demonstrated that the developed formulations are practically non-irritant, further studies using corneal epithelial cell lines and in vivo models are necessary to comprehensively evaluate cytotoxicity and long-term ocular safety, particularly considering the known membrane activity of AmphB and bile salts. In addition, accelerated stability testing (40 ± 2 °C/75 ± 5% RH, 4 weeks) demonstrated preservation of fiber morphology and FTIR profiles, supporting the physicochemical stability of the developed system under stress conditions.

Taken together, these findings demonstrate that γ-CD and STC-enhanced AmphB-loaded PVA electrospun fibers constitute an innovative platform that combines amorphous drug dispersion, rapid dissolution, and acceptable tolerability, with potential to improve patient compliance and therapeutic outcomes in fungal keratitis.

While Fourier transform infrared spectroscopy (FTIR) and X-ray diffraction (XRD) confirmed the formation of an amorphous solid dispersion and the absence of drug–polymer incompatibility, solid-state NMR could provide complementary molecular-level insight into the inclusion complexation between AmphB and γ-cyclodextrin, as well as detailed information on drug–polymer interactions within the submicron fiber matrix. Although this technique was beyond the scope of the present formulation-focused study, it represents a valuable direction for future structural characterization.

Despite these promising results, further investigations are required to evaluate ocular pharmacokinetics, in vivo therapeutic efficacy, and long-term safety. In addition, the specific role of sodium taurocholate should be further elucidated to optimize the balance between permeation enhancement and ocular safety. Ultimately, clinical evaluation will be necessary to confirm the translational potential of this nanofiber-based ophthalmic delivery system.

## Figures and Tables

**Table 1:** Composition of amphotericin B-loaded (AmphB-loaded) precursor solutions. Samples prepared from AmphB/gamma-cyclodextrin (γ-CD)/polyvinyl alcohol (PVA)/sodium taurocholate (STC).

Formulation Code	PVA (% (*w*/*w*))	AmphB (% (*w*/*w*))	γ-CD (% (*w*/*w*))	STC (% (*w*/*w*))
F1	12	0.03	5	1
F2	12	0.03	5	2
F3	12	0.03	5	3
F4	12	0.03	7.5	1
F5	12	0.03	7.5	2
F6	12	0.03	7.5	3
F7	12	0.03	10	1
F8	12	0.03	10	2
F9	12	0.03	10	3

**Table 2:** Present kinetic parameters of amphotericin B-loaded (Amph B- loaded) nanofibers. Note: M∞ values slightly exceeding 100% represent minor analytical overshoot within acceptable experimental error (±5%) and confirm complete drug release.

Formulation Code	M_∞_	β Parameter	τ_d_	Correlation Coefficient (R^2^)
F1	100.83 ± 0.31	0.6675	2.03	0.9976
F2	101.66 ± 0.36	0.6151	2.06	0.9991
F3	103.21 ± 0.75	0.4931	1.8	0.9971
F4	103.00 ± 0.70	0.4644	1.53	0.9976
F5	101.15 ± 1.62	0.4848	1.69	0.9887
F6	100.93 ± 0.58	0.6417	1.8	0.9852
F7	103.31 ± 1.31	0.5017	1.92	0.9938
F8	100.69 ± 1.34	0.4827	1.5	0.9878
F9	104.16 ± 1.35	0.4573	1.86	0.9944

**Table 3:** Summarizes the zone of inhibition (ZOI) results from the agar diffusion assay, comparing the activity of amphotericin B-loaded (AmphB-loaded) nanofibers with AmphB solution in Dimethyl sulfoxide (DMSO) against *Candida albicans*, *Fusarium solani*, and *Aspergillus fumigatus*.

Fungal Strain	Nanofiber 2.5 mg (ZOI in mm)			Nanofiber 5 mg (ZOI in mm)			AmphB in DMSO (ZOI in mm)		
	24 h	48 h	72 h	24 h	48 h	72 h	24 h	48 h	72 h
*Candida albicans*	22 mm	22 mm	-	23 mm	23 mm	-	25 mm	24 mm	-
*Aspergillus fumigatus*	23 mm	23 mm	-	24 mm	23 mm	-	26 mm	25 mm	-
*Fusarium solani*	-	12 mm	11 mm	-	13 mm	12 mm	-	16 mm	14 mm

## Data Availability

The original contributions presented in this study are included in the article/Supplementary Material. Further inquiries can be directed to the corresponding authors.

## Supplementary Materials

The following supporting information can be downloaded at https://www.mdpi.com/article/10.3390/pharmaceutics18040464/s1. Figure S1: Solubility of amphotericin B (AmphB) in water as a function of increasing gamma-cyclodextrin (γ-CD) concentration; Table S1: Chemical stability of Amphotericin B (AmphB) aqueous solution containing gamma-cyclodextrin (γ-CD), measured over various time intervals following heat treatment; Table S2: Average fiber diameters of AmphB-loaded electrospun nanofibers with their respective skewness and kurtosis values; Figure S2: Fiber diameter distributions of amphotericin B-loaded (AmphB-loaded) nanofibers. Samples prepared from AmphB/gamma-cyclodextrin (γ-CD)/polyvinyl alcohol/sodium taurocholate (STC). All samples contained PVA (12% *w*/*w*) and AmphB (0.03% *w*/*w*). F1–F3: 5% γ-CD with 1–3% STC; F4–F6: 7.5% γ-CD with 1–3% STC; F7–F9: 10% γ-CD with 1–3% STC; Figure S3: Agar plates showing inhibition zones around Amphotericin B-loaded (AmphB-loaded) nanofibers and Amph B solution in Dimethyl sulfoxide (DMSO) for *Candida albicans*, *Aspergillus fumigatus*, and *Fusarium solani*. The inhibition zones were measured to compare the efficacy of the two formulations.

## Abbreviations

The following abbreviations are used in this manuscript:

AmphB	Amphotericin B
ASD	Amorphous solid dispersion
CAM	Chorioallantoic membrane
CDs	Cyclodextrins
DMSO	Dimethyl sulfoxide
EDTA	Ethylenediaminetetraacetic acid
FTIR	Fourier transform infrared spectroscopy
HET-CAM	Hen’s Egg Test on Chorioallantoic Membrane
PBS	Phosphate-buffered saline
PVA	Polyvinyl alcohol
SEM	Scanning electron microscopy
STC	Sodium taurocholate
XRD	X-ray diffraction
ZOI	Zones of inhibition
γ-CD	Gamma-cyclodextrin
