# Supplementary material for: Development and Formulation of Nanofiber-Based Ophthalmic Inserts for the Treatment of Fungal Keratitis
Source: Pharmaceutics. 2026 Apr 10;18(4):464. doi: 10.3390/pharmaceutics18040464 (PMC13118770; doi:10.3390/pharmaceutics18040464)
Supplement: Supplementary file 1 [file pharmaceutics-18-00464-s001.zip › pharmaceutics-4219648-supplementary.pdf]

Development and Formulation of Nanofiber-based Ophthalmic Inserts for the Treatment of Fungal Keratitis

Safaa Omer [1], Nándor Nagy [2], Júlia Pongrácz [3], Bence Dávid Tóth [4], Balázs Pinke [5], László Mészáros [5], Katalin Kristóf [3], Adrienn Kazsoki [1,*] and Romána Zelkó [1,*]

[1] University Pharmacy Department of Pharmacy Administration, Semmelweis University, Hőgyes Endre Street 7-9, H-1092 Budapest, Hungary; safaa.omer@phd.semmelweis.hu
[2] Department of Anatomy, Histology and Embryology, Semmelweis University, Tűzoltó Street 58, H-1094 Budapest, Hungary; nagy.nandor@semmelweis.hu
[3] Department of Laboratory Medicine, Faculty of Medicine, Semmelweis University, Üllői út 26, H-1085 Budapest, Hungary; pongracz.julia@semmelweis.hu (J.P.); kristof.katalin@semmelweis.hu (K.K.)
[4] Department of Pharmaceutics, Semmelweis University, Hőgyes Endre Street 7, H-1092 Budapest, Hungary; toth.bence@semmelweis.hu
[5] Department of Polymer Engineering, Faculty of Mechanical Engineering, Budapest University of Technology and Economics, Műegyetem Rkp. 3, H-1111 Budapest, Hungary; pinke.balazs.gabor@gpk.bme.hu (B.P.); meszaros@pt.bme.hu (L.M.)
* Correspondence: kazsoki.adrienn@semmelweis.hu (A.K.); zelko.romana@semmelweis.hu (R.Z.); Tel.: +36-302-060-093 (A.K.); +36-208-259-621 (R.Z.)

3. Results and discussion

3.1. Solubility study of amphotericin B (AmphB)/gamma-cyclodextrin (γ-CD)

A phase solubility diagram was constructed by plotting the molar concentration of dissolved AmphB against the concentration of γ-CD (**Figure S1**).

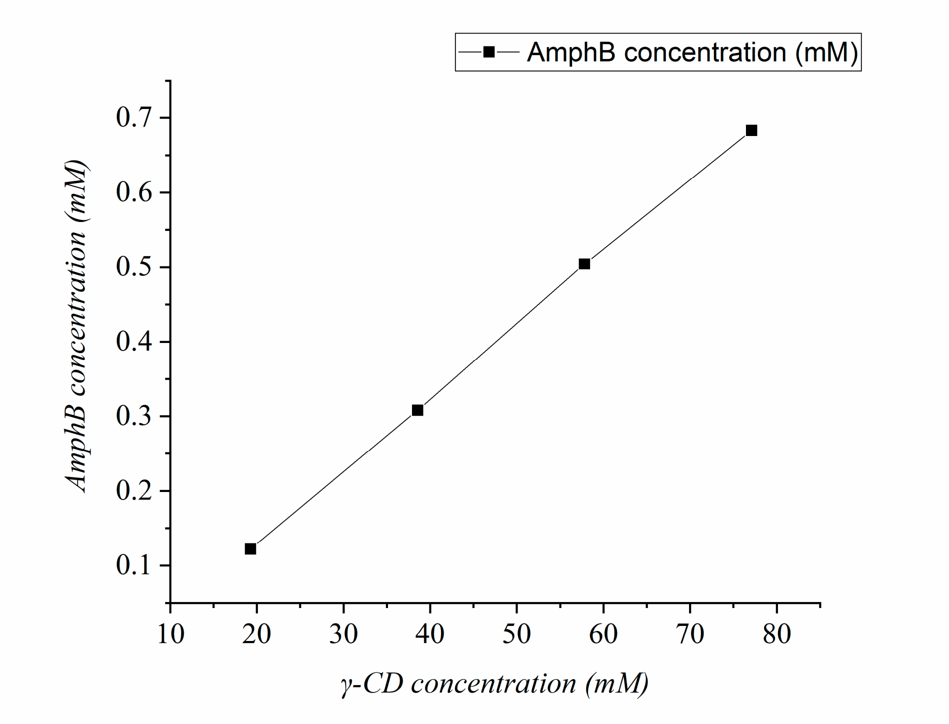

Figure S1. Solubility of amphotericin B (AmphB) in water as a function of increasing gamma-cyclodextrin (γ-CD) concentration.

3.2. Chemical stability of amphotericin B (AmphB)

The chemical stability of AmphB, when complexed with γ-CD, was assessed by subjecting the complex to an elevated temperature of 60°C for 0, 30, 60, and 120 minutes (**Table S1**). The content of AmphB was then analyzed using UV-Vis spectroscopy. Temperature stability studies revealed that γ-CD helps maintain AmphB stability at both room and elevated temperatures. The results demonstrate that γ-CD enhances the temperature stability of AmphB. The protective effect of γ-CD is likely due to its ability to encapsulate AmphB within its hydrophobic cavity, shielding the drug from direct exposure to heat. This encapsulation restricts the mobility of AmphB molecules, thereby reducing the likelihood of thermal degradation reactions such as oxidation, hydrolysis, or structural rearrangement. The stabilization effect observed is consistent with previous studies on other drug-cyclodextrin complexes, where cyclodextrins have been shown to enhance the thermal and chemical stability of encapsulated drugs [1].

Table S1. Chemical stability of Amphotericin B (AmphB) aqueous solution containing gamma-cyclodextrin (γ-CD), measured over various time intervals following heat treatment.

Time of exposure at 60°C (min)	AmphB concentration (μg/mL) (Mean ± SD)
0 min	10.96 ± 0.037
30 min	10.89 ± 0.07
60 min	10.87 ± 0.027
120 min	10.83 ± 0.047

3.3. Morphological characterization

The values of skewness and kurtosis reveal that the distribution curves of electrospun AmphB-loaded samples are of normal and skewed shapes (**Table S2**).

Table S2. Average fiber diameters of AmphB-loaded electrospun nanofibers with their respective skewness and kurtosis values

Formulation code	γ-CD (% (w/w)	Average fiber diameter (nm) ± SD (nm)	Skewness	Kurtosis
F1	5	267 ± 43	-0.0557	-0.3030
F2	5	237 ± 36	-0.1183	-0.4918
F3	5	216 ± 33	-0.0339	-0.4026
F4	7.5	239 ± 29	-0.1196	-0.1718
F5	7.5	278 ± 38	-0.2634	0.0042
F6	7.5	284 ± 38	0.0771	-0.8489
F7	10	310 ± 35	-0.4573	0.1026

F8	10	282 ± 35	0.0859	-0.7378
F9	10	299 ± 40	-0.1081	-0.7700

The results indicate that different solutions' compositions produced different distribution curves, including homogenous normal distribution and compound distribution curves (**Figure S2**).

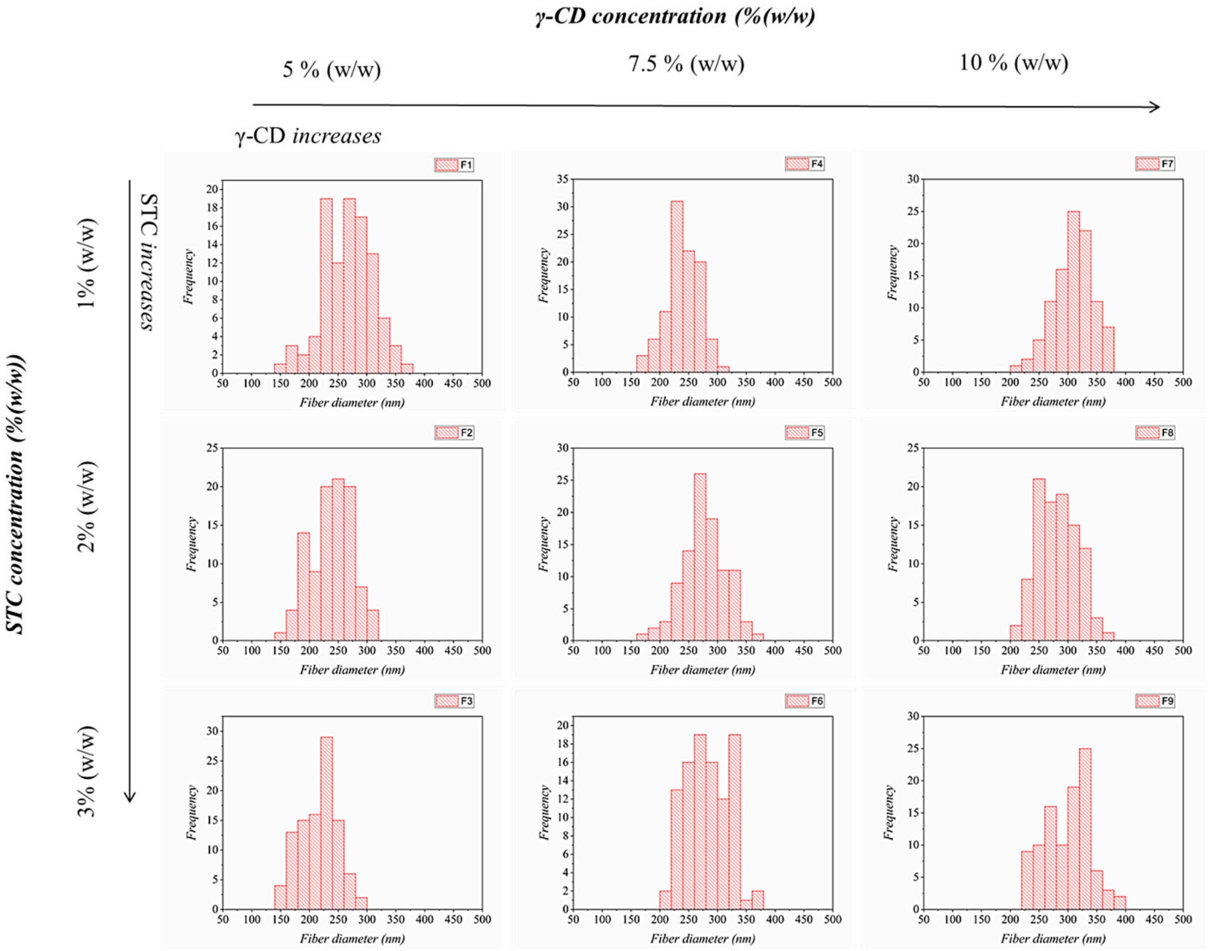

Figure S2. Fiber diameter distributions of amphotericin B-loaded (AmphB-loaded) nanofibers. Samples prepared from AmphB/gamma-cyclodextrin (γ-CD)/polyvinyl alcohol/sodium taurocholate (STC). All samples contained PVA (12% w/w) and AmphB (0.03% w/w). F1–F3: 5% γ-CD with 1–3% STC; F4–F6: 7.5% γ-CD with 1–3% STC; F7–F9: 10% γ-CD with 1–3% STC.

3.7. Amphotericin B nanofiber activity against Candida albicans, Fusarium solani, and Aspergillus fumigatus

3.7.1. Agar diffusion assay

Figure S3 illustrates the agar diffusion assay results, where the inhibition zones around the AmphB-loaded nanofiber and AmphB solution disks are shown for each fungal pathogen.

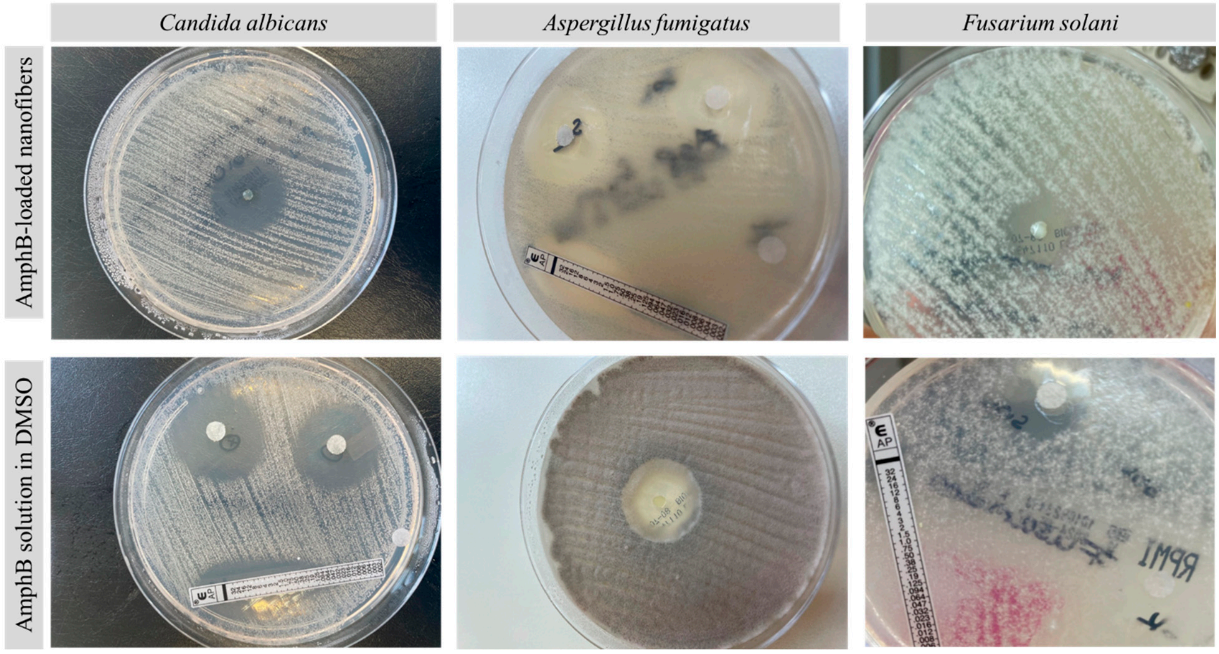

Figure S3. Agar plates showing inhibition zones around Amphotericin B-loaded (AmphB-loaded) nanofibers and Amph B solution in Dimethyl sulfoxide (DMSO) for *Candida albicans*, *Aspergillus fumigatus*, and *Fusarium solani*. The inhibition zones were measured to compare the efficacy of the two formulations

References

1. Nath, L.; Laldinchhana; Choudhury, A.D.; Barakoti, H.; Devi, C.M. Development and Validation of UV-Vis Spectrophotometric Method for Estimation of Amphotericin B. *Research Journal of Pharmacy and Technology* **2020**, *13*, 55–59.
